# Nasopalatine Duct Cyst with Impacted Inverted Mesiodens: A Rare Case Report and Literature Review

**DOI:** 10.1155/2022/5981020

**Published:** 2022-12-19

**Authors:** Shamimul Hasan, Deepika Bablani Popli, Syed Ansar Ahmad, Keya Sircar, Shahnaz Mansoori, Kirti Dua

**Affiliations:** ^1^Department of Oral Medicine and Radiology, Faculty of Dentistry, Jamia Millia Islamia, New Delhi, India; ^2^Department of Oral Pathology, Faculty of Dentistry, Jamia Millia Islamia, New Delhi, India; ^3^Department of Oral and Maxillofacial Surgery, Faculty of Dentistry, Jamia Millia Islamia, New Delhi, India; ^4^Faculty of Dentistry, Jamia Millia Islamia, New Delhi, India

## Abstract

Nasopalatine duct cyst (NPDC) is a non-odontogenic, developmental epithelial cyst that accounts for 1% of maxillary cysts. It often arises due to the spontaneous proliferation of the epithelial tissue remnants, although trauma, bacterial infection, and mucous retention may also trigger the proliferation. Owing to its slow-growing, asymptomatic nature, the cyst is often discovered as an accidental finding during routine clinical and radiographic examinations. However, the majority of cases present as a tiny, asymptomatic swelling just posterior to the palatine papillae. Radiographically, it appears as a well-defined oval or round radiolucency in the maxillary anterior teeth region and should be differentially diagnosed with inflammatory periapical lesions and a wide incisive foramen. A pulp vitality test is essential to rule out lesions of endodontic origin. Microscopically, NPDCs display a mixed pattern of the epithelial lining and exhibit neurovascular bundles (small to medium-sized nerves, arteries, and veins), and minor salivary glands in the cystic connective tissue, a distinctive feature facilitating a confirmatory diagnosis. Enucleation and marsupialization remain the treatment of choice. NPDC associated with impacted mesiodens is an extremely uncommon entity. A comprehensive literature search carried out on the PubMed and Google Scholar search engines revealed only three cases of NPDC with impacted mesiodens to date. The purpose of this study is to report an extremely rare case of NPDC associated with an impacted inverted mesiodens in a 19-year-old male patient who presented with an asymptomatic swelling in the maxillary anterior teeth region. To the best of our knowledge, this is only the fourth reported case of NPDC with impacted mesiodens.

## 1. Introduction

Supernumerary teeth refer to an extra tooth as compared to normal dentition, with a reported prevalence rate of 0.3–0.8% and 0.5–3.8% in the primary and permanent dentitions, respectively [1].

The precise etiopathogenesis of supernumerary teeth is still debatable, although trauma, drugs, infections, radiation, genetic, environmental, and hormonal factors may serve as potential triggers that might have an impact on tooth development. Phylogenetic reversion (Atavism) theory, dental lamina hyperactivity theory, and dichotomy theory are the different recommended theories in etiopathogenesis [2, 3]. Various syndromic conditions (cleidocranial dysplasia, Gardner's syndrome, cleft lip and palate, oral-facial-digital syndrome, and chondroectodermal dysplasia) may also be associated with supernumerary teeth [4].

Studies have demonstrated that panoramic radiographs are inaccurate in the identification of supernumerary teeth due to a narrow image layer in the maxillary anterior region and spinal superimposition [5, 6]. About 50% of supernumerary teeth might go undetected on the panoramic radiographs; hence, a higher level of dental training and more accurate diagnostic aids are imperative for detecting the precise location of supernumerary teeth and their relation with the contiguous vital structures [6].

Localization of supernumerary teeth is cardinal to ascertain the surgical technique and also to avert the potential iatrogenic injury to the adjacent anatomic structures during the surgical procedure [7]. Published literature has demonstrated that various radiographic techniques, including the horizontal tube shift technique (HTST) and vertical tube shift technique (VTST), have been employed to locate supernumerary teeth [8, 9]. However, there is no scientific affirmation to validate the relative accuracy of HTST and VTST for the exact localization of supernumerary teeth. Mallineni et al. analyzed the relative accuracy of these two techniques and affirmed that the VTST is more precise in the localization of unerupted supernumerary teeth in the maxillary anterior region [7]. Recently, artificial intelligence models using deep learning (DL) algorithms with convolutional neural networks have been developed for the detection of mesiodens on panoramic radiographs. A study by Kuwada et al. focused on the automated detection of mesiodens in permanent dentition using DL algorithms [10]. However, a study by Ha et al. focused on the automated detection of mesiodens on panoramic radiographs for primary, mixed, and permanent dentition groups [11].

Supernumerary teeth may be associated with an array of complications, such as delay/failure of permanent teeth eruption, rotation/displacement/impaction/crowding and dilacerations of adjacent permanent teeth, and mid-line diastema. Resorption of the adjacent tooth roots, dentigerous cyst, and ameloblastoma development has also been reported with supernumerary teeth [3, 12]. Dentigerous cysts in association with supernumerary teeth account for 5–6% of all dentigerous cysts, of which about 90% have accompanying mesiodens [2].

Nasopalatine duct cyst (NPDC) is a development, non-odontogenic, epithelial cyst that constitutes 1% of all maxillary cysts [13]. Meyer in 1914 was the first to describe it as an accessory paranasal sinus [14]. NPDC is now the favored term for the synonymous “incisive canal cyst” as it may be seen within the nasopalatine canal (NPC) or in the palatal soft tissues or at the canal opening [15].

NPDC associated with impacted mesiodens is an extremely uncommon entity. A comprehensive literature search carried out on the Google Scholar and PubMed search engines revealed only three case reports of NPDC with impacted mesiodens [16–18]. The purpose of this study is to report an extremely rare case of NPDC associated with an impacted inverted mesiodens in a 19-year-old male patient who presented with asymptomatic swelling in the maxillary anterior teeth region. To the best of our knowledge, this is only the fourth reported case of NPDC with impacted mesiodens.

## 2. Case Description

**Figure 1:**
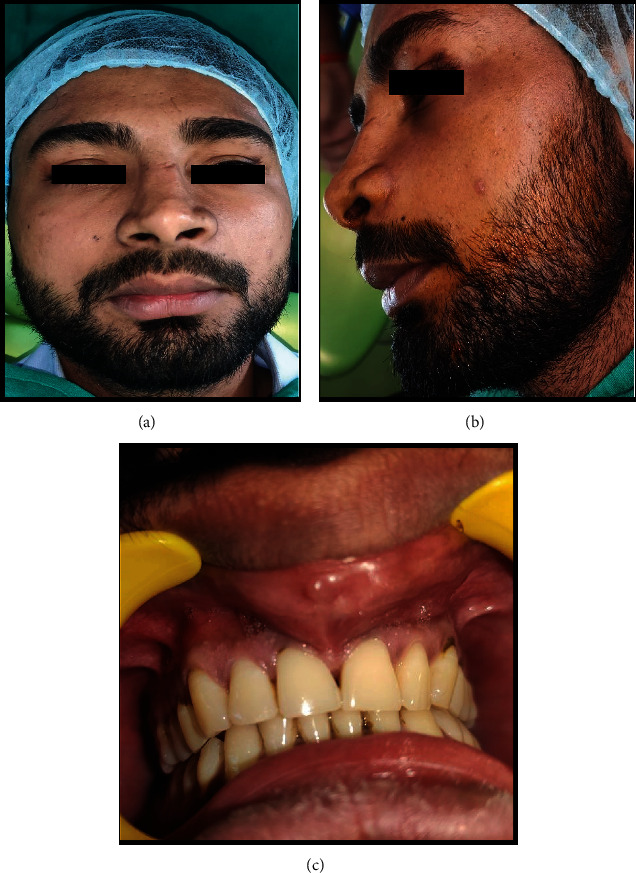
(a) and (b) Extraoral swelling on the left mid-facial region. (c) Intraoral swelling in the maxillary anterior region.

A 19-year-old male patient presented with a slow-progressing, painless swelling in the maxillary anterior teeth region for the last 4 months. The initially smaller swelling has gradually increased with time to reach the present size. The past medical and family anamnesis was non-contributory, with no history of preceding trauma. The patient consulted a private practitioner one month back, who prescribed him antibiotics and analgesics for 7 days (tablet Augmentin 625 mg twice daily and tablet Ketorol DT 10 mg twice daily). However, the swelling persisted, and the patient visited our outpatient department. On physical examination, a diffuse swelling roughly 5cm × 5cm in size, involving the left mid-facial region, was seen. The swelling extended up to 2 cm below the left infra-orbital margin to about 2 cm inferior to the lower aspect of the upper lip with a slight elevation of the upper lip and anteroposteriorly extended from the right nasal alae to about 3 cm anterior to the left tragus of the ear. A slight superior deviation of the left nasal alae with left nasolabial fold obliteration was also noticeable. Palpatory findings elicited a non-tender, firm-hard consistency swelling with no increase in the surface temperature [Figures 1(a) and 1(b)]. On intraoral examination, a solitary, well-demarcated, 5cm × 4cm sized swelling in the maxillary anterior vestibule region with labial cortex expansion was appreciated. The swelling extended from the distal margin of tooth #12 to the mesial aspect of tooth #14 anteroposteriorly. Superiorly, the swelling obliterated the labial mucobuccal fold and inferiorly extended up to the gingival teeth margins. Palpatory features were suggestive of a non-tender swelling, with peripheral firm consistency and central fluctuations [Figure 1(c)]. The dentition showed normal tooth count and color with no clinically carious or missing teeth.

**Figure 2:**
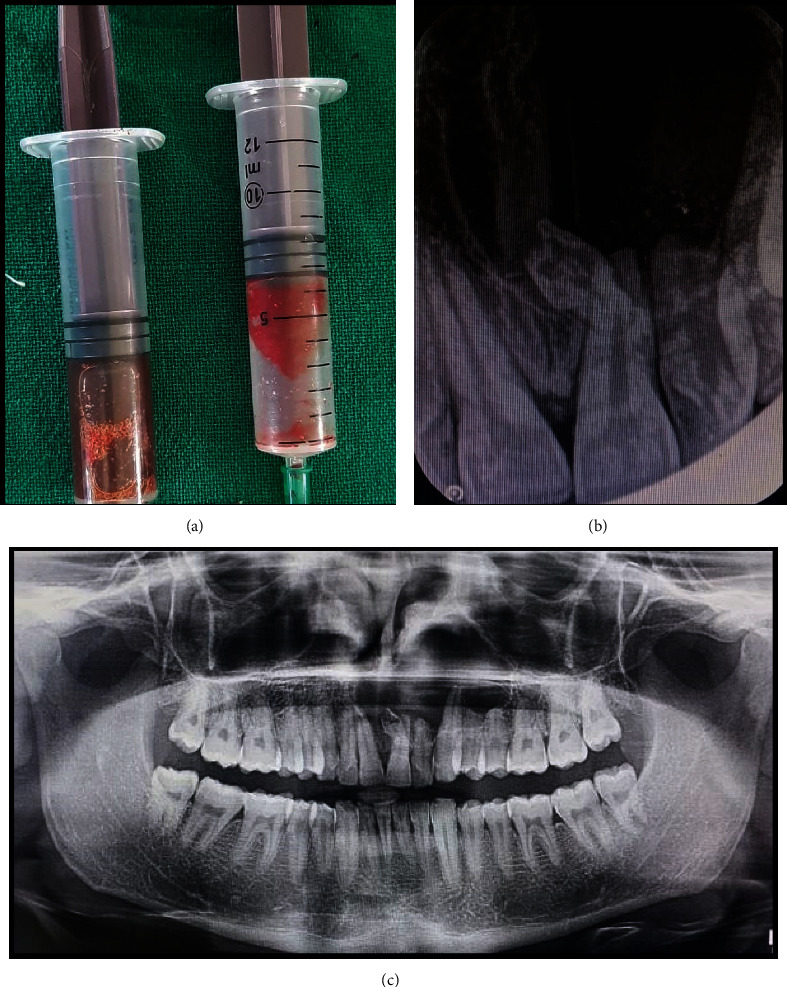
(a) Fine-needle aspiration revealed yellow-brown, blood-tinged cystic aspirate. (b) and (c) IOPAR and OPG demonstrate a well-defined unilocular radiolucency with an impacted inverted mesiodens in the maxillary anterior region.

The maxillary anterior teeth elicited a positive response (representing a vital pulp tissue) with pulp vitality tests. Fine-needle aspiration yielded a brownish-yellow, blood-tinged cystic aspirate [Figure 2(a)]. An intraoral periapical radiograph (IOPAR) revealed a well-demarcated, heart-shaped unilocular radiolucency, peripherally encircled with a thin corticated border, and an impacted inverted mesiodens in the anterior maxillary region. The radiolucent lesion extended mesiodistally from the distal aspect of tooth #11 to the mesial margin of tooth #23 and inferiorly up to the root apices of the maxillary incisors. Root resorption of teeth #21 and #22 with distal flaring of the roots of teeth #11 and #21 was also noticed [Figure 2(b)]. Orthopantomogram (OPG) showed a well-circumscribed, ovoid, unilocular radiolucent lesion with peripheral corticated borders in the maxillary anterior region. The radiolucency extended from the mesial aspect of tooth #12 to the distal margin of tooth #25 and encircled the pericoronal aspect of the impacted inverted mesiodens. The floor of the left maxillary sinus was slightly pushed superiorly, and a slight distal displacement of the roots of teeth 11 and 21 with root resorption of teeth #21, #22, #24, and #25 was also apparent [Figure 2(c)].

Cone-beam computed tomography (CBCT) was advised to ascertain the extent of the lesion and three-dimensional (3D) location of the mesiodens and their relation with the adjacent vital structures. However, the patient denied the radiographic investigation due to financial constraints; thus, the exact localization of the mesiodens and its relationship with the lesion and adjacent structures could not be ascertained. Hence, in the absence of a CBCT scan, it is likely that the patient had a cystic lesion and also a mesiodens without a relationship between them.

Considering the patient's age, location, and radiographic features, radicular cyst, dentigerous cyst, and adenomatoid odontogenic tumor were considered in the differential diagnosis.

**Figure 3:**
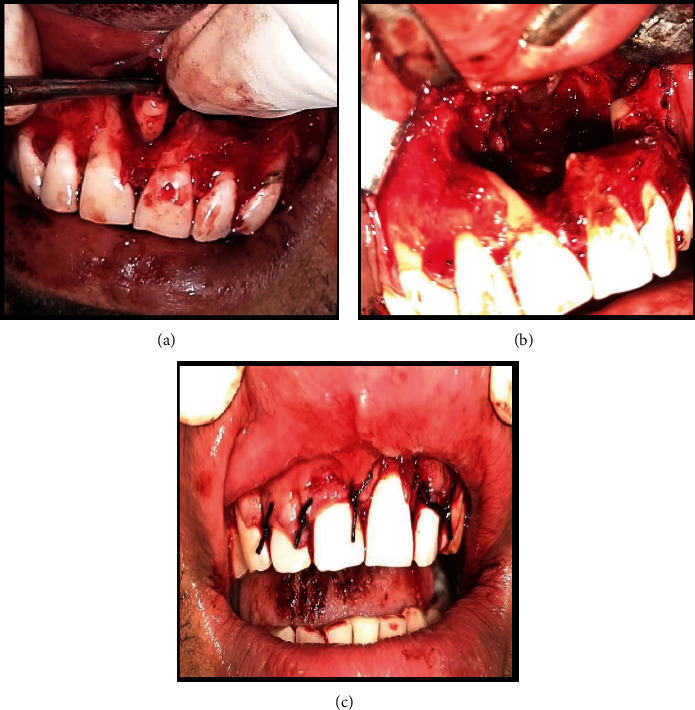
(a)–(c) The surgical enucleation of the lesion.

Surgical enucleation of the lesion was considered (after an unremarkable hematological evaluation and obtaining written patient consent). A crevicular incision from the right maxillary canine to the left second molar was made, and the flap was reflected. Eight milliliters of blood-tinged, straw-colored fluid was aspirated from the cystic cavity, and impacted mesiodens was seen between the two maxillary central incisors. The impacted mesiodens was attached to the bone and not adherent to the cystic lining. Carnoy's solution was used to completely remove the cystic lining. Iodoform gauze was inserted in the cavity, and one end of the gauze was taken out from below the alae of the nose. Sutures were placed, and the enucleated specimen was sent for histopathologic evaluation [Figures 3(a), 3(b), and 3(c)].

**Figure 4:**
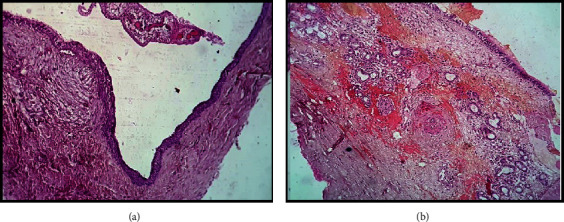
(a) and (b) Epithelial lining showing a combination of pseudostratified ciliated columnar and nonkeratinized stratified squamous epithelium varieties, with mucous glands and neurovascular bundles in the cystic wall (H&E stain, 100×).

The submitted hemotoxylin and eosin (H&E) sections (100×) showed a cystic epithelial lining with a fibrous connective tissue wall. The epithelial lining displayed a combination of epithelial varieties with the dominant type being ciliated pseudostratified columnar epithelium with goblet cells. Non-keratinized stratified squamous epithelium and primitive flat epithelium resembling reduced enamel epithelium were also present in places. Few areas show prominent juxta-epithelial hyalinization. The cystic wall revealed numerous mucous acini, muscular arteries, small veins, and nerves. Inflammatory cells along with cholesterol clefts and giant cells were also noted in some sections. These microscopic features were suggestive of a NPDC [Figures 4(a) and 4(b)].

There was uneventful postoperative healing, and no recurrences were noted during the 3-month-periodic follow-up.

## 3. Discussion

Mesiodens occurring in the maxillary anterior tooth region is the commonest reported supernumerary teeth, with a prevalence of 0.15–1.9%. [19]. They describe a common developmental deformity in mixed dentition [20]. Based on their appearance, mesiodens can be classified as rudimentary mesiodens and supplementary mesiodens in permanent and deciduous dentition, respectively. They are termed conical, tuberculate, or molariform based on their morphology [21].

Generally, mesiodens has no evident manifestations and only present as an increase in the tooth number [22]. However, mesiodens can give rise to numerous complications related to both the adjacent teeth and other vital structures. The likely dental complications with mesiodens include crowding with delayed permanent incisors eruption, midline diastema, rotation/displacement of the permanent incisors, caries, periodontitis, occlusal discrepancies, root resorption/abnormal root formation of the adjacent teeth, and infection. Mesiodens may also exert an impact on the neighboring vital anatomical structures, causing NPC /nasal floor perforation or the development of cysts [19].

Mesiodens is usually identified as incidental findings on routine radiographic investigations or in cases where the tooth fails to erupt normally [19]. Published literature has appraised various techniques for the localization of mesiodens. Conventional radiographic techniques, such as periapical radiographs, maxillary occlusal, and panoramic radiographs, have been employed for mesiodens diagnosis, localization, and management in the past. However, due to the imprecise localization of the mesiodens, they fail to fulfill the requirements of mesiodens extraction under the defined treatment protocol [23, 24]. The anterior maxillary and mandibular regions frequently pose a diagnostic challenge due to a narrow focal trough, vertebral superimposition, and the air space between the palate and the tongue, thus questioning the precise localization of the mesiodens with panoramic radiographs [11]. Although, the same lingual opposite buccal (SLOB) radiographic technique has demonstrated some diagnostic utility, however, the loss of image clarity, magnification, and superimposition limits the use of plain two-dimensional (2D) radiographs. Hence, it is imperative to delineate the 3D location of the mesiodens for a precise diagnosis and treatment plan [25, 26]. CBCT can ascertain not only the 3D location, shape, and axial inclination of mesiodens but also their relationship with adjacent vital structures. Over the years, CBCT has become an indispensable preoperative radiographic investigation for mesiodens localization and management [27].

In the present case, only 2D imaging (IOPAR and panoramic radiography) was carried out and revealed a well-defined unilocular radiolucency with impacted inverted mesiodens. However, 3D imaging (CBCT) was imperative in this case to ascertain the precise extent of the lesion and 3D location of the mesiodens, and their relation with the adjacent vital structures. CBCT could not be carried out as the patient was not willing due to financial constraints.

A surgical extraction is the primary treatment of mesiodens, and repositioning it in the dental arch may be another treatment option. However, the timing of extraction of unerupted mesiodens is still debatable [23]. Most authors advocate early intervention and surgical extraction of the unerupted mesiodentes as soon as they are identified [28].

Hence, an impacted mesiodens should be extracted at a young age when it appears to cause injury to the adjacent tissues. The optimal age for mesiodens extraction has been documented to be 6–7 years, after which more complications may be anticipated [29]. Other authors propose that extraction of the impacted mesiodens should be delayed until the root formation of the adjacent permanent incisor tooth is completed. This may avert the potential loss of vitality or root resorption of the adjacent permanent teeth. Moreover, early surgical intervention in a young patient necessitates the treatment under general anesthesia, which be associated with potential complications and might create psychological dental anxiety in the child [30]. In addition, early exposure and bonding of the impacted incisor may result in supporting bone loss and formation of scar tissue, thus further preventing the tooth eruption [31]. However, extraction is not always the mainstay of treatment for supernumerary teeth. Unerupted mesiodentes that do not appear to cause potential damage to the adjoining dentition and structures are routinely monitored without undergoing extraction [23, 24, 28].

The NPC, also referred to as the incisive canal or anterior palatine canal, is generally seen in the midline, just posterior to the maxillary central incisors. The anterior oral opening and the nasal opening of NPC are known as incisive foramen (IF) and Stenson's foramina, respectively [32]. NPC connects the oral and nasal cavities by linking the IF and the nasal foramen [33].

The content of the NPC comprises a neurovascular bundle (formed by the nasopalatine nerve, the terminal branches of the nasopalatine artery, and the veins) encircled by the connective tissue, fat, and seromucous glands [34]. Another anatomical structure called the nasopalatine duct may occasionally be seen within the NPC. The nasopalatine duct generally exhibits gradual degeneration; hence, it is now regarded as a vestigial structure in humans, embryologically and phylogenetically [35].

The etiopathogenesis of these cysts is still ambiguous, although some researchers have suggested that NPDCs arise from the spontaneous proliferation of the epithelial tissue remnants [13, 35–38]. Trauma (ill-fitting dentures/orthodontic appliances, previous endodontic treatment, or implant placement), bacterial infection, or mucous retention may also act as triggering factors for the proliferation of the epithelial tissue remnants [13, 37, 38]. As these cysts occur in incisive canals of the human fetus, spontaneous cystic degeneration of epithelial remnants is also proposed [13, 37].

However, in our case, there was no history of trauma, and the clinical evaluation did not exhibit periodontal pockets in the maxillary anterior teeth region. Hence, cystic development in our case could not be attributed to any factor other than spontaneous proliferation.

NPDCs associated with impacted mesiodens are an extremely uncommon entity. A detailed literature search was carried out on the Google Scholar and PubMed search engines using the following keywords: NPDC, supernumerary teeth, impacted teeth, and mesiodens. Case reports published in the English language up to August 2022 were extensively searched. This comprehensive literature search finally revealed only three cases of NPDC with impacted mesiodens, as summarized in Table 1 [16–18].

NPDC is one of the many gnathic pathologies but is distinctive in that it is site-specific (seen in only the midline of the maxillary anterior region) [36, 39]. NPDCs generally exhibit an age predilection (frequently seen in the age range of 40–60 years), although it may affect any age group [36, 37, 39–41]. However, age should not be a criterion to rule out its occurrence in the younger age group, as a case of NPDC has been documented in a 7-year-old child [35, 42]. NPDCs usually exhibit a male predilection (M : F 3 : 1) [43, 44].

Our patient was a 19-year-old male who reported a slow-progressing, painless swelling in the maxillary anterior teeth region for the past 4 months.

Owing to their slow-growing asymptomatic nature (40% of cases), they are usually observed as incidental findings during regular clinical and radiographic investigations [13, 17, 37, 38, 40, 43, 44]. A tiny asymptomatic swelling just behind the palatine papilla is the presenting symptom in most cases; however, a few cases may present a midline swelling on the labial aspect of the alveolar ridge. Larger cysts may cause severe destruction and exhibit “through and through” fluctuation between the palatal and labial cortex [17, 36, 37]. The size of the swelling is usually <2 cm in diameter. However, larger and more aggressive lesions up to 52 mm may cause complete perforation of the labial and palatal cortex [13, 45]. The superficial cyst usually presents as a fluctuant swelling with a bluish hue, in contrast to the deeper cysts covered by masticatory mucosa, unless ulcerated. Few cases of nasopalatine nerve impingement may cause numbness and tingling sensations [13, 17, 36, 37]. Depending on the discharge, the patient may experience either a salty taste (in cases of mucoid discharge) or a foul taste (in cases of purulent discharge). A cyst with mesiodens and tooth displacement as seen in the present case is a rarity [17, 36, 37].

Radiographically, NPDC generally exhibits a well-circumscribed, unilocular midline radiolucency, with an oval/round shape [35–37, 40, 43]. However, a few cases may present as a heart-shaped lesion, either due to notching by the nasal septum or superimposition of the nasal spine on the radiolucent area [36, 37, 43]. Divergence of the central incisor roots is a frequent radiographic feature of NPDCs, although root resorption is an infrequent occurrence. Superior displacement of the nasal floor has also been reported [46]. Computed tomography not only renders superior delineation of the lesion and the contiguous anatomical structures but also facilitates the best surgical treatment strategy [17, 36, 37, 44].

Radiographic appearance in our case was in sync with the previously published literature. Radiographically, a well-circumscribed unilocular radiolucent lesion with corticated borders, encompassing an impacted inverted mesiodens, was seen in the maxillary anterior region. Root resorption of the involved teeth, with a distal displacement of central incisor roots and superior displacement of the floor of the left maxillary sinus, was also noticed.

CBCT could not be performed as the patient was not willing due to financial constraints.

Microscopically, NPDCs display mixed patterns of epithelial linings. Ciliated columnar epithelium and squamous epithelium predominate the nasal floor and the oral cavity region, respectively, and the intermediate region is lined by the cuboidal epithelium [36, 37, 45]. NPDC is the only cyst in the oral and maxillofacial regions that exhibit neurovascular bundles (small to medium-sized nerves, arteries, and veins) and minor salivary glands in the cystic connective tissue. This distinctive histopathological feature facilitates a confirmatory diagnosis [41].

Our case demonstrated a combination of epithelial varieties, with the ciliated pseudostratified columnar epithelium in predominance. Numerous mucous acini, muscular arteries, small veins, nerves, and inflammatory cells were also appreciated microscopically.

Inflammatory lesions, such as periapical cysts and granulomas [37, 40, 41, 44, 46], wide IF [9, 36, 37, 41, 44–46], odontogenic keratocyst [40], nasolabial cyst [40, 47], and adenomatoid odontogenic tumor [48], may be given a place in the differential diagnosis of NPDCs. The radiographic appearance of a periapical cyst/granuloma with a maxillary central incisor tooth often simulates that of an asymmetric NPDC. However, a periapical cyst usually involves the periapical region of non-vital teeth with a discontinuity of the lamina dura. NPDCs typically appear as well-delineated, round/ovoid/pear/heart-shaped radiolucencies in between the roots of a vital maxillary central incisor with a continuous lamina dura. In addition, a shift in the horizontal angulation alters the position of an NPDC, whereas the periapical cyst sustains its location around the maxillary central incisors periapex [17, 37, 44, 46]. In such situations, the pulp vitality test is essential to rule out lesions of endodontic origin [17, 18, 37, 40, 43].

A positive pulp vitality test with the associated teeth was seen in the present case, thus negating any periapical inflammatory lesion.

NPDCs may be clinically differentiated from a widened IF by aspiration of the lesion. An ill-defined radiolucency with a diameter of 0.6–0.8 cm in the maxillary central incisor region is often considered a wide IF. On the other hand, NPDCS usually appear as well-circumscribed, spherical radiolucent lesions, often with the superior cystic margin at a higher position than that of an IF [36, 37, 44].

Odontogenic keratocysts often exhibit an aggressive growth pattern and appear as ostelytic lesions with defined borders in the inter radicular/periapical areas of maxillary anterior teeth. However, they are mostly seen as multilocular radiolucent lesions in the mandibular posterior teeth [40]. Furthermore, microscopically, the cystic lumen is composed of a “cheesy” material, often with a parakeratinized epithelium lining [48].

Nasolabial cysts are soft tissue cysts and do not exhibit bone involvement, hence not apparent on the radiographic examination. In addition, the nasolabial cysts most commonly occur in the nasolabial sulcus, lateral to the midline [40, 47].

Adenomatoid odontogenic tumors most commonly occur in the maxillary anterior teeth region of young females. The radiolucency extends apically beyond the cementoenamel junction. In addition, these lesions display a mixed radiolucent-radiopaque radiographic pattern [48].

Enucleation, marsupialization, and decompression are the frequently employed treatment regimens. NPDCs are generally managed with enucleation, although marsupialization is recommended for large aggressive cystic lesions [18]. Cystic aspiration is generally performed before surgical enucleation. Surgical enucleation has a favorable prognosis and low recurrence rates. [35]. Malignant transformation of NPDCs is an extremely uncommon complication [41].

In our case, the lesion was surgically enucleated, and uneventful postoperative healing was observed. There was no recurrence during the 3-month follow-up period.

The main limitation in this case report was that a CBCT scan could not be performed due to the lack of facility and the patient's unwillingness due to financial constraints. Hence, a 3D extent of the lesion, the precise localization of the mesiodens, and its relation with the contiguous structures could not be ascertained. The 2D radiographic imaging (IOPAR and panoramic radiography) performed in this case was not sufficient to ascertain the 3D localization of the mesiodens and its relationship with the cystic lesion and adjacent vital structures.

## 4. Conclusion

Mesiodens seen in the maxillary anterior tooth region is the most frequently occurring supernumerary teeth. However, NPDCs in association with mesiodens are an extremely uncommon finding with only three reported cases to date. This study aims to report a case of NPDC with an impacted inverted mesiodens. Surgical enucleation was carried out and favorable healing with no recurrences was observed during the 3-month follow-up.

## 5. Recommendations

- A 3D radiographic examination, such as CBCT, is essential to delineate the exact shape, orientation, and location of the mesiodens, and also to evaluate their relationship with the contiguous vital structures.
- NPDCs should also be given a place in the differential diagnosis of the radiolucent lesions in the anterior maxillary region.
- Pulp vitality should be carried out to exclude the lesions of endodontic origin and ensure precise diagnosis and treatment strategy.

## 6. Patient Feedback

The patient experienced uneventful favorable healing after the enucleation of the cystic lesion and was completely satisfied with the treatment protocol. The patient has been carefully examined and followed up for 3 months, during maintenance visits scheduled every 4 weeks. The patient did not report any signs of recurrences and was completely asymptomatic during the follow-up period.

## Figures and Tables

**Table 1:** Reported NPDC cases with impacted mesiodens.

S.no.	Author(s)	Age (years)/sex	Clinical findings	Radiographic features	Treatment plan
1.	Gopal et al. [16]	18/M	Swelling and recurrent pus discharge in teeth #12–#22	Impacted inverted mesiodens within a well-circumscribed unilocular radiolucency	Surgical enucleation and removal of the mesiodens
2.	Tariq Salamm et al. [17]	55/F	Pain and swelling in teeth #12–#21	Impacted supernumerary tooth within a heart-shaped radiolucency	Surgical enucleation and removal of the mesiodens
3.	Bereket and Kaynar [18]	27/M	Swelling in the anterior palatal region of teeth #15–#25	Horizontally located supernumerary tooth within a lytic lesion in the anterior maxilla	Cystic decompression followed by surgical enucleation
